# The Potential Role for Cognitive Training in Sport: More Research Needed

**DOI:** 10.3389/fpsyg.2018.01121

**Published:** 2018-07-03

**Authors:** Courtney C. Walton, Richard J. Keegan, Mike Martin, Harry Hallock

**Affiliations:** ^1^School of Psychology, University of Queensland, Brisbane, QLD, Australia; ^2^Brain and Mind Centre, The University of Sydney, Sydney, NSW, Australia; ^3^University of Canberra Research Institute for Sport and Exercise, Faculty of Health, University of Canberra, Canberra, ACT, Australia; ^4^New South Wales Institute of Sport, Sydney, NSW, Australia; ^5^Berlin School of Mind and Brain, Humboldt-Universität zu Berlin, Berlin, Germany; ^6^Department of Neurology, Charité – Universitätsmedizin Berlin, Berlin, Germany

**Keywords:** cognitive training, performance enhancement, cognition, athletes, sport

## Abstract

Sports performance at the highest level requires a wealth of cognitive functions such as attention, decision making, and working memory to be functioning at optimal levels in stressful and demanding environments. Whilst a substantial research base exists focusing on psychological skills for performance (e.g., imagery) or therapeutic techniques for emotion regulation (e.g., cognitive behavioral therapy), there is a scarcity of research examining whether the enhancement of core cognitive abilities leads to improved performance in sport. Cognitive training is a highly researched method of enhancing cognitive skills through repetitive and targeted exercises. In this article, we outline the potential use of cognitive training (CT) in athlete populations with a view to supporting athletic performance. We propose how such an intervention could be used in the future, drawing on evidence from other fields where this technique is more fruitfully researched, and provide recommendations for both researchers and practitioners working in the field.

## The Role of Cognition in Sport

The role of cognition and neuroscience in understanding, predicting, and potentially improving elite sports performance is an area that has received increased interest in recent years (Yarrow et al., 2009; Walsh, 2014; Katwala, 2016). This notion is validated by studies showing that athletes perform faster and more accurately on specific cognitive tasks (Mann et al., 2007; Voss et al., 2010). Such findings have been supplemented by studies showing that baseline cognitive ability is able to predict future sporting achievement (Vestberg et al., 2012, 2017; Mangine et al., 2014).

Given the above evidence, the aim of this paper is to introduce some of the considerations in this potentially booming field of practice, incorporating knowledge of cognitive training (CT) in other cohorts. We highlight that further research is needed before we can reliably inform coaches, athletes, and support staff of any potential benefits from this technique. Well planned studies which incorporate collaborative interdisciplinary knowledge are needed to progress this field most rapidly.

## A Brief Introduction to Cognitive Training

Computerized CT is a flourishing field of research [and commercial business (George and Whitehouse, 2011)] within the scope of cognitive enhancement, with applications being studied extensively in many different cohorts. The central focus of CT is to target specific cognitive functions, through repetitive computerized exercises. Complexity and response time demands change frequently during and across sessions, in accordance with changes in individual performance as to avoid over- or under-stimulation.

Cognitive training has shown efficacy in terms of post-training performance on cognitive testing, assumed to represent an improved capacity in the specific domain (i.e., near transfer), though relevant to this discussion, also on aspects of motor functions such as gait (Smith-Ray et al., 2015; Walton et al., 2018). Improvements in cognition have been shown in those with neurodegenerative disease, along with other psychiatric and neurological disorders (Keshavan et al., 2014; Lampit et al., 2014b; Leung et al., 2015; Hallock et al., 2016; Hill et al., 2016; Motter et al., 2016).

Despite many positive findings for CT on cognition, it must be acknowledged that there is a strong and healthy debate surrounding overall efficacy, justifiably, given the claims from some commercial companies often outweigh the underlying scientific evidence (e.g., see the well documented exchange between researchers^1^) and extensive review by Simons et al. (2016). Additionally, the CT field has struggled in general from high levels of methodological heterogeneity amongst studies, a poor ability to define improvement in a functional capacity, and small sample sizes (Walton et al., 2014). In the current context, it is also worth noting that CT has predominantly shown most promise in populations characterized by deficits in cognition, in that it has primarily been used to *raise* what may have previously decreased, or reduce further losses. As illustrated above, elite athletes may actually have superior functioning within specific domains, and thus it is currently unknown whether CT can *enhance* cognitive performance in this sample.

## Enhancing Cognition for Elite Performance

Anecdotal evidence suggests that exercises which resemble CT are already being implemented in sports environments. Indeed, there are many companies now selling software aimed to deliver this very product (e.g., *NeuroTracker, Axon Sports*). As researchers and advocates of CT, it is encouraging to see the enthusiastic uptake of the technology in new settings. However, it appears the bulk of existing evidence regarding CT’s efficacy, on which athletes and coaches must currently rely, comes from direct claims delivered by some of the commercial companies themselves (or their sponsored athletes), which often do not appear backed up by peer-reviewed accessible science. The early stages of CT research more generally were once in a similar state, however, the field now sees hundreds of publications per year (Walton et al., 2014), progressively fine-tuning facets of design. Nevertheless, given that CT is not a ‘one size fits all’ intervention, our knowledge of what does, doesn’t, or could work for these specific sporting purposes lags significantly behind other cohorts (Harris et al., 2018). This must change before these interventions are to be wholeheartedly endorsed and promoted.

Harris et al. (2018) reviewed the evidence for real-world transfer of effects using commercially available CT interventions. These authors found only one study (Romeas et al., 2016) to have been completed within a sporting context, illustrating the lack of evidence for CT in athletes. This study employed 3-dimensional multiple object tracking (3D-MOT), a task which challenges users to keep track of multiple moving objects in a dynamic and changing visual field. Intuitively, this skill has implications for sports performance where athletes must be able to accurately process, for example, multiple teammates, the opposition, obstacles and targets all at once. Athletes have been shown to excel in this task, with Faubert (2013) showing that professional athletes across multiple sports have a higher baseline ability to perform this task, but also faster learning curves than non-elite athletes, and non-athletes. Romeas et al. (2016) examined the training in 19 male soccer players over three groups (3D-MOT, passive and active control). The experimental group trained twice weekly for 5 weeks, while the active control watched 3D soccer videos accompanied by short interviews based on decision making, thus reinforcing the expectation of training benefit to the athletes. Following training, the intervention group improved by 15% in a measure of on-field passing decision-making, in addition to subjective confidence levels in decision-making accuracy. There were not improvements in shooting or passing accuracy, which again reflects the potential constraints on transferring of CT benefits to related-but-different tasks.

There were limitations to this work, not least that the intervention group only included seven athletes (two dropped out). It must also be acknowledged that this study was conducted by researchers who are, ostensibly, heavily invested in the tool; providing further evidence that navigating the realm of combining scientifically rigorous studies with financially lucrative tools will be inherently difficult (Rabipour and Raz, 2012; Simons et al., 2016). This potential conflict-of-interest has previously been a common criticism of CT, where some companies who have enormous financial incentives to show positive results have been involved in the research studies which seek to objectively determine efficacy. While we certainly suggest no wrongdoing whatsoever, and the author’s conflicts of interest were clearly provided, we would like to highlight that separating proof of efficacy research studies from those invested in the outcomes is always preferential (Ahn et al., 2017).

Separately, and not reviewed by Harris et al. (2018), Hirao and Masaki (2018) used the Simon Task to make those trained more able to shoot toward the opposite direction of a goal-keepers initial lateral movement. Twenty-nine lacrosse players were split into two groups, either conducting Stimulus-Response Compatibility Training, or an active control. In line with the authors’ hypothesis, the intervention group shot to the opposite side of the goalie’s movement more often than the active control post-training, though this did not lead to more goals being scored, potentially due to poor shooting velocity. Additionally, though there was a significant difference between groups at post-test, the treatment group did not show a significant improvement from baseline. It is also worth noting that the treatment group performed the cognitive task less accurately at follow up, and significantly worse than the control group following training. Therefore, while this study is interesting and has some well-designed elements, we cannot obtain a full picture of the training efficacy and theoretical underpinnings for the improvements found in this work.

The work of Romeas et al. (2016) and Hirao and Masaki (2018) are certainly exciting, and a positive step in the right direction for investigating the potential efficacy of CT in sport via peer-reviewed controlled trials. However, given the known difficulty of achieving far transfer following CT, it is surprising that the only known studies have both provided positive effects. Replication is required before such results can be relied upon, and of particular importance, publication of null results in similar studies is encouraged so as to minimize creating a biased literature.

Of note, there are studies which have examined other techniques of training which also incorporate some cognitive-perceptual ability (see review by Hadlow et al., 2018). By contrast, these studies have been more focused around aspects including: (a) video-based training that is highly specific to the outcome (e.g., quickly predicting the direction of a batsman’s strike from video (Hopwood et al., 2011); (b) computer-based putting training (Fery and Ponserre, 2001); or (c) making decisions faster than ‘real-time’ on sporting scenarios (Lorains et al., 2013; Farahani et al., 2017). While this work is very interesting and likely has great potential for investigating the role of cognition in increasing performance, we do not consider this to be CT per se, but rather an alternative method of sport-specific *practice* that involves computerized tools. By definition, CT should target specific cognitive functions that are not simply reflective of the desired outcome. Given this, when discussing CT for sport, we are specifically interested in the act of improving core cognitive processes which in turn fundamentally underlie sports performance.

## How Do We Determine Training Efficacy?

One of the most complex aspects in applying cognitive enhancement to athletes is how to best determine efficacy. This problem is not specific to the sporting context, however, and is an issue mirrored in other cohorts. For example, though CT shows relatively consistent improvements in cognitive performance during testing in older adults, the effects on activities of daily living or the likelihood of subsequent dementia development are not well established, despite these arguably being the more important outcomes (Jones, 2018). In athletes, an improvement in post-CT neuropsychological testing is interesting, but not a practically meaningful result for athlete or coach. What is needed is evidence that the intervention has lifted the level of performance relevant to the sport in question and beyond a practically meaningful threshold. Notably, however, in the current age of ‘marginal gains’, it is difficult to constitute what reflects meaningful improvement in the eyes of sports organizations. Given that a noted criticism of CT is the current lack of consistent evidence for far-transfer, extra care must be given to how efficacy following CT is measured, as this absence of far transfer could be a result of insensitive testing as opposed to ineffective training. In this section we will briefly discuss this issue.

Unfortunately, accurately determining an immediate follow-up outcome is difficult. Sport is highly variable with many unique and interrelated contributors to performance (e.g., nutrition, mental state, injuries, sleep disturbance, teammate and opposition performance, weather conditions, and natural performance variability, etc.); meaning that using one-off performances as a marker of change is troublesome. Furthermore, simply finding objective indices of sporting performance – particularly in interactive sports and team sports – is famously problematic. Assessing changes in more prolonged timescales, such as season performance (Vestberg et al., 2012; Mangine et al., 2014) is perhaps the preferential end goal, however, again, so many variables predict this performance throughout the season, that it is very hard to determine precisely the unique impact that CT has had.

Assessing performance in more controlled sporting environments is one way to get around the problems posed by measuring sporting performance [i.e., Romeas et al. (2016) above]. In these tests, specific sporting skills – which rely on more cognitive aspects such as decision-making, game-based working memory, and reaction time – can be assessed by scorers who are blinded to the condition athletes received (Smith et al., 2007; Romeas et al., 2016). Another way of simplifying the complex problem could be combining physical and cognitive measures into hybrid tests, potentially using a virtual reality (VR) environment.

This approach would allow for testing parameters to remain constant, and furthermore be adaptive to the athlete’s abilities, preventing potential ceiling effects. For example, to test reaction time, current computerized neuropsychological testing may ask the subject to press a key as quickly as possible upon seeing a specific stimulus. An on-field measure of reaction time could be the time it takes to initiate movement after seeing an object (e.g., goalkeeper reacting to a penalty kick). In a VR environment, reaction time could be tested by asking the athlete to catch a moving object and measuring both the initial movement and overall time lapsed. Unlike the on-field example, here the speed, location and trajectory of the object can be controlled, and unlike the neuropsychological example this is sport-specific. The stimuli can be easily adapted to the athlete’s abilities and also to various sports. Furthermore, it can be adapted to further test an athlete’s reaction time in specific situations, such as whilst under physical fatigue. This illustrates the innovative potential of VR in validly assessing post-training changes, and also the potential for a new holistic approach of training and testing paradigms for athletes. However, as discussed by Miles et al. (2012) there are still many fundamental concepts within VR that require further research.

## Designing Cognitive Training Studies in Sport

As researchers with varied experience in CT across different cohorts, in addition to working with athletes in elite settings, we hope to be able to give some suggestion on some of the elements CT research should strive for. **Table 1** highlights some important considerations moving forward. We note that these are in many way personal reflections, given that we currently do not have the evidence base to accurately determine what is appropriate in a sports context (Harris et al., 2018).

**Table 1:** Considerations for future CT studies targeting athletic performance.

CT trial design suggestions	CT intervention components to explore further	CT intervention outcomes to explore further
- *Trial design*: CONSORT guidelines to be followed. -*Control groups*: An active control which has minimal negative impact on athletes and teams is required to create equal expectancy bias. -*CT delivery*: Supervised training 2–3 times a week, for approximately 40 minutes to an hour each session. - *Multi-domain training*: Little to no evidence for cognitive domain transfer. Multi-domain training recommended, targeting multiple cognitive abilities specific to the sport of interest. - *Multisite Studies*: Both CT and sports science studies are notoriously underpowered. As recruitment is difficult, multiple sites could be used to maximize *n*. -*Funding and Conflicts of Interest*: If studies are funded by CT companies, care must be taken to ensure impartiality.	- *Training environment*: Is CT more beneficial when integrated with a physical task, under fatigue, employing sport-specific virtual reality, or supplemented with neural stimulation? - *Opportunity costs*: CT must not take away from regular physical training/coaching. At what dose is CT most complementary to regular training? - *Gamification and motivation*: Investigation as to what strategies make CT more engaging/effective such as educating athletes on cognition, competition of results, virtual reality etc.	- *Sport performance*: CT must impact more than simply cognition to be relevant. What are the best measures of efficacy in athletes? - *Additional outcomes:* Neuropsychological, questionnaire, and neuroimaging markers can all be useful to understand improvements, particularly if correlated to any sport-related gains. - *Head injury*: Can CT play a role in athletes recovering from head injuries? If undertaken prior to head injury, could CT negate associated cognitive decline?


**FIGURE 1:**
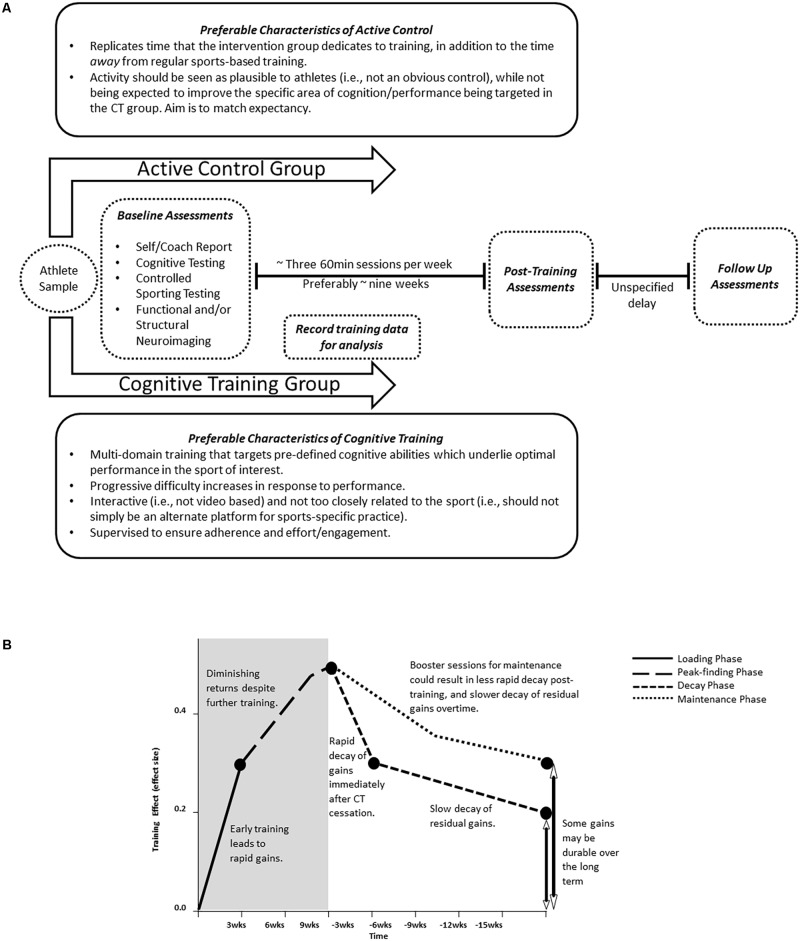
**(A)** Example RCT design to asses CT for athletes. Brief outline of the preferable characteristics of the training and control groups, duration and frequency of the intervention and the types of assessments that should be administered. **(B)** Assumed therapeutic effect of CT overtime. Training (gray) initially produces rapid gains during the loading phase, which then begin to plateau during the peak-fining phase. Once training is ceased (white), during the decay phase, gains decay rapidly and then gradually over time. However, if a maintenance phase comprising of booster sessions is implemented during the decay phase, then gains may be durable over a longer period of time. In any case, training will result in a higher level of cognition when compared to baseline. Image adapted from Figure 3 in Lampit et al. (2014a).

**Figure 1A** illustrates an example CT design that may be of interest to researchers hoping to undertake CT interventions in athletes. Based on work from Lampit et al. (2014b), we propose training of both groups should aim for three 45–60-min sessions a week, for roughly 9 weeks. Training below these recommendations may prove to be ineffective due to insufficient time for synaptic plasticity to occur, whilst overtraining could be ineffective due to fatigue or disengagement. **Figure 1B** based on data from Lampit et al. (2014a) shows a hypothesized timecourse of CT efficacy, illustrating the potential phases of training. We can see that eventually (at the end of peak-finding phase) the effect will begin to plateau, perhaps due to overtraining, and it is here where it’s opportunity cost may begin to waver. However, these two figures are hypotheses based on original and meta-analytical findings and cannot truly be known in a sports-specific population until more studies have been conducted.

There is currently an ongoing debate as to the scientific merit of employing active control groups over passive control groups in the wider literature. Nevertheless, evidence exists that variables other than the CT intervention, most notably expectations bias, can significantly influence post-training performance (Foroughi et al., 2016), and thus expectations regarding any perceived benefit of training between groups must be well accounted for within the trial design. Given the infancy of the field as applied to sport, we suggest the use of active control groups is crucial. While matching the time that intervention groups spend dedicated to CT, in addition to any expectancy effects, sports cohorts provide an additional element. Given that athletes dedicate significant amounts of time to structured training, it is possible that those involved in a CT research study may spend less time on regular physical training, and that time must also be matched in the active control group to avoid influencing the outcome of performance.

To promote a transfer between physical and CT, a novel approach to CT could be to conduct it within a VR environment, however, a high level of caution is required to be clear that any improved performance is not simply reflective of practice effects. In using VR environments that combine physical and CT, there is also the potential ability to improve athletes’ ‘resilient cognition’ (Keegan, 2017): that is, their ability to maintain near-optimal cognitive performance and thus decision making despite physical fatigue. This reflects another novel use of CT in athletes, which is to not just improve cognition underlying performance, but additionally, to improve cognitive performance under specific physical demands.

Recent changes to gamification of CT have been instrumental to improving engagement of these training programs. Lumsden et al. (2016) have illustrated how gamification can be effective, including by increasing participant motivation, long-term engagement, and to increase ecological validity. However, methodological concerns with only a small body of work mean any conclusions are tentative. Nevertheless, CT in athletes could potentially be most useful when gamification principles are employed to maximize motivation, perhaps via aspects such as training exercises being sport-related and implementing competitive aspects.

As discussed, ideal outcomes are undefined as of yet, and we propose multiple measures may be best including athlete and blinded-coach assessment, cognitive testing, a controlled sports-specific assessment as discussed and neuroimaging where resources allow. The sustainability of effects in CT is also not well understood (Lampit et al., 2014a), but it is likely that less-frequent ongoing training is needed for continued benefits after a more intensive loading phase (Rebok et al., 2014). Where feasible, follow-up testing could provide valuable information on the maintenance of improvement.

## Conclusion

We suggest that there is a significant gap in our knowledge-base regarding how CT can be implemented to improve athletes’ performance. Given the link between cognition and sporting ability, there is a clear rationale for further investigating whether CT could benefit athletes. However, the current evidence-base means that we cannot know whether this tool is effective, and given the difficulties achieving far transfer in other cohorts, we caution around investing too heavily in such methods at this point in time. We do, however, recognize there is merit to investigating further, and research that would develop this understanding will require the assistance of coaching staff and athletes to establish high quality studies, with the ultimate aim of better understanding how these methods could help athletes maximize every potential for their performance.

## Author Contributions

CW and HH conceptualized the manuscript. CW prepared the original draft. All authors edited and gave final approval for publication and were accountable for this work.

## Conflict of Interest Statement

The authors declare that the research was conducted in the absence of any commercial or financial relationships that could be construed as a potential conflict of interest.

## Footnotes

1 https://www.cognitivetrainingdata.org/the-controversy-does-brain-training-work/response-letter/
